# Studying cerebral hemodynamics and metabolism using simultaneous near-infrared spectroscopy and transcranial Doppler ultrasound: a hyperventilation and caffeine study

**DOI:** 10.14814/phy2.12378

**Published:** 2015-04-23

**Authors:** Runze Yang, Julien Brugniaux, Harinder Dhaliwal, Andrew E Beaudin, Misha Eliasziw, Marc J Poulin, Jeff F Dunn

**Affiliations:** 1 Department of Radiology, Faculty of Medicine, University of CalgaryCalgary, Alberta, Canada; 2 Faculty of Medicine, Hotchkiss Brain Institute, University of CalgaryCalgary, Alberta, Canada; 3 Department of Clinical Neurosciences, Faculty of Medicine, University of CalgaryCalgary, Alberta, Canada; 4 Department of Physiology and Pharmacology, Faculty of Medicine, University of CalgaryCalgary, Alberta, Canada; 5 Department of Public Health and Community Medicine, Tufts University School of MedicineBoston, Massachusetts, USA; 6 Libin Cardiovascular Institute of AlbertaCalgary, Alberta, Canada

**Keywords:** Brain, near-infrared spectroscopy, transcranial Doppler ultrasound

## Abstract

Caffeine is one of the most widely consumed psycho-stimulants in the world, yet little is known about its effects on brain oxygenation and metabolism. Using a double-blind, placebo-controlled, randomized cross-over study design, we combined transcranial Doppler ultrasound (TCD) and near-infrared spectroscopy (NIRS) to study caffeine's effect on middle cerebral artery peak blood flow velocity (Vp), brain tissue oxygenation (S_t_O_2_), total hemoglobin (tHb), and cerebral oxygen metabolism (CMRO_2_) in five subjects. Hyperventilation-induced hypocapnia served as a control to verify the sensitivity of our measurements. During hypocapnia (∼16 mmHg below resting values), Vp decreased by 40.0 ± 2.4% (95% CI, *P* < 0.001), while S_t_O_2_ and tHb decreased by 2.9 ± 0.3% and 2.6 ± 0.4%, respectively (*P* = 0.003 and *P* = 0.002, respectively). CMRO_2_, calculated using the Fick equation, was reduced by 29.3 ± 9% compared to the isocapnic-euoxia baseline (*P* < 0.001). In the pharmacological experiments, there was a significant decrease in Vp, S_t_O_2_, and tHb after ingestion of 200 mg of caffeine compared with placebo. There was no significant difference in CMRO_2_ between caffeine and placebo. Both showed a CMRO_2_ decline compared to baseline showing the importance of a placebo control. In conclusion, this study showed that profound hypocapnia impairs cerebral oxidative metabolism. We provide new insight into the effects of caffeine on cerebral hemodynamics. Moreover, this study showed that multimodal NIRS/TCD is an excellent tool for studying brain hemodynamic responses to pharmacological interventions and physiological challenges.

## Introduction

Cerebral hemodynamics (blood flow, metabolic rate, oxygenation) are important brain physiological parameters that can change in response physiological challenge or drug ingestions (Blaha et al. 2007; Chen and Parrish 2009; Bain et al. 2013). Near-infrared spectroscopy shows promise as a tool for investigating microvascular oxygenation and hemoglobin saturation (Yang et al. 2013). By combining NIRS with transcranial Doppler, we propose that new information can be obtained about the impact of respiratory or drug challenges on brain physiology.

Caffeine is a widely used psychostimulant that is present in many food and drinks, with ∼54% of adults drinking coffee every day (Field et al. 2003). However, not much is known about the impact of caffeine on brain oxygenation, cerebral blood volume (CBV), and cerebral metabolic rate for oxygen (CMRO_2_). Caffeine is a stimulant that is able to enhance cognition and promote alertness (Pilli et al. 2013), suggesting it increases neuronal activity and metabolic rate. At the same time, caffeine is known to significantly decrease cerebral blood flow (CBF) (Perthen et al. 2008; Chen and Parrish 2009; Vidyasagar et al. 2013). This appears paradoxical to its stimulating effects, as a decrease in CBF is usually associated with a decrease in CMRO_2_ (Buxton et al. 1998; Kida et al. 2007).

The hemodynamic effects of caffeine are caused by nonspecific antagonism of both the adenosine A_1_ and A_2A_ receptors (Pelligrino et al. 2010). Activation of adenosine A_1_ receptors will restrict presynaptic glutamate release, causing an overall inhibitory effect (Stone et al. 2009). Activation of A_2A_ receptors will activate protein kinase A (PKA) and cause intracellular calcium influx into the vascular smooth muscle cells, causing vasodilation (Ngai et al. 2001). By antagonizing these receptors (i.e., using caffeine), vasoconstriction will occur and there will be increases in neuronal activity. Due to the opposite effects of adenosine A_1_ and A_2A_ receptors, measurement of both CBF and brain oxygenation must be made in order to generate a more complete picture of caffeine's effect on the brain.

Similar to caffeine, the impact of hypocapnia on cerebral blood flow has been well documented, as CO_2_ is a potent vasodilator. As such, mild hypocapnia has been explored in the context of concussion to help decrease the intracranial pressure (Alexander et al. 2013). However, limited data exist on the effects profound hypocapnia (ΔPetCO_2_ >10 mmHg) on cerebral oxygenation and metabolic rate. Understanding the hemodynamic of profound hyperventilation may provide insight into the regulation of brain metabolism under extreme conditions.

CBF through the large vessels within the Circle of Willis can be monitored noninvasively using transcranial Doppler ultrasound (TCD). TCD is sensitive to changes in blood flow velocity in large blood vessels, such as the middle cerebral artery (MCA), and has been widely used to monitor cerebral blood flow in a variety of disorders (Gulyas et al. 2001; Bonoczk et al. 2002). Because of its low cost and excellent temporal resolution, TCD is an ideal instrument to monitor pharmacologically induced CBF changes.

Brain oxygenation can be measured noninvasively using near-infrared spectroscopy (NIRS). NIRS uses the principles of the Beer–Lambert law and the different absorption spectra of oxyhemoglobin (HbO_2_) and deoxyhemoglobin (HHb) to determine their respective concentrations within microvessels and tissues of the brain (Fantini et al. 1999). Most commercially available NIRS systems are only capable of measuring changes from a baseline and not the absolute concentration of hemoglobin. In contrast, a frequency-domain NIRS (fdNIRS) system provides the ability to calculate absolute hemoglobin concentrations (Fantini et al. 1999). Thus, with fdNIRS two important parameters, total hemoglobin (tHb) (HbO_2_+HHb) and tissue oxygenation (S_t_O_2_) (HbO_2_/tHb) of the brain can be calculated. tHb is related to the cerebral blood volume (CBV) while S_t_O_2_ is an indicator of the brain oxygenation (Yang et al. 2013).

Both TCD and NIRS are relatively inexpensive, noninvasive instruments with good temporal resolution. In addition, they have great synergies, as they measure different aspects of the hemodynamic response of the brain. Therefore, in this study we combined TCD and NIRS to generate an enhanced picture of caffeine-induced hemodynamic changes in the brain. We hypothesized that caffeine impacts both brain hemodynamics and CMRO_2,_ and that the combination of TCD and NIRS will be sensitive in detecting these changes.

## Materials and Methods

### Study participants

We recruited nine subjects. Their mean age was 31 years old (SD 9, range 20–50). This study was approved by the Conjoint Health Research Ethics Board at the University of Calgary.

### Study design and data collection

This was a double-blind, placebo-controlled, randomized, cross-over study conducted over two different days. Subjects ingested 200 mg of caffeine on 1 day and 200 mg of lactose placebo on the other. For all conditions, TCD was used to monitor right MCA CBF and fdNIRS was used to monitor frontal lobe microvascular hemoglobin levels.

On both experimental days, prior to pharmacological ingestion, subjects followed a constant pattern of hyperventilation in accordance with an audio alarm for 5 min. Dynamic-end tidal forcing (Poulin et al. 1996) was used to maintain the end tidal partial pressure of CO_2_ (PetCO_2_) at approximately 22 mmHg by adjusting the fraction of inspired CO_2_ levels on a breath-by-breath basis during the hyperventilation period. We collected TCD and fdNIRS data for 5 min prior to hyperventilation, 5 min during hyperventilation, and for 5 min after hyperventilation during a recovery period. During baseline and recovery, the inspired gas was maintained at isocapnic euoxia. PetO_2_ levels were maintained throughout the hyperventilation paradigm at approximately 85 mmHg. Analysis of the data was performed using the middle 3 min for each phase of the hyperventilation challenge (baseline, hyperventilation, recovery). This paradigm can also serve as a control to determine if TCD and fNIRS were sensitive enough to detect an exercise that is known to change cerebral blood flow and brain oxygenation.

For the pharmacological study, TCD and fdNIRS data were collected for 5 min before the ingestion of the drug (caffeine or placebo), and then continuously for 70 min after consumption of caffeine or placebo. Results were examined at pre-drug ingestion, 30 min post drug ingestion, and 60 min post drug ingestion.

### Near-infrared spectroscopy

The ISS OxiplexTS is a commercially available fdNIRS system and was used in this study. The ISS calculates hemoglobin concentrations by the frequency-domain method (Fantini et al. 1999). The NIRS probe used in this study was supplied by ISS. The probe consists of one detector and four fiber optic sources with a minimum separation of 2.0 cm, and a maximum separation of 3.2 cm from the detector. The NIRS probe emits light at 690 and 830 nm into the tissue, with a modulated frequency of 110 MHz.

Before collecting data, the machine was warmed up for 30 min. The system was then calibrated using a phantom with known absorption and scattering coefficients, and the calibration was checked using another phantom with different absorption and scattering coefficients. After calibration, the NIRS probe was placed on the right forehead as close to the hairline as possible and secured in place with Velcro straps.

### Transcranial Doppler ultrasound

Cerebral blood flow was assessed by recording the peak velocity of the blood traveling through the MCA (Vp) using a 2-MHz pulsed TCD (PCDOP 842; SciMed, Bristol, UK). The probe was held in place with a snug-fitting headgear (marc600, Spencer Technologies, Seattle, WA). The techniques and procedures used in this study have been described in detail elsewhere (Poulin et al. 1996; Ide et al. 2003, 2007).

### Respiratory gases

Respired gas was sampled continuously via a catheter held within a nostril via a modified nasal cannula and monitored for the fraction of carbon dioxide and oxygen with a mass spectrometer (AMIS 2000, Innovision, Odense, Denmark). Arterial oxyhemoglobin saturation (S_a_O_2_) was monitored using a pulse oximeter (3900p; Datex-Ohmeda, Madison, Wisconsin).

### Multimodal data processing

We combined the Vp data from TCD, S_t_O_2_ data from NIRS as well as S_a_O_2_ data from the pulse oximeter to calculate a relative CMRO_2_ (rCMRO_2_) using the Fick principle 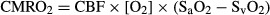 (Brown et al. 2003). Where S_a_O_2_ is the arterial saturation and S_v_O_2_ is the venous saturation.

S_t_O_2_ is comprised of an unknown proportion of venous blood, arterial blood, and capillary blood; for simplicity, we assumed that the NIRS data represented a weighted mean of the arterial and venous data. By this assumption, S_t_O_2_ is composed of arterial and venous blood, and the percent contribution of S_a_O_2_ and S_v_O_2_ to S_t_O_2_ must add to 100%. As a result, S_t_O_2_ can be represented using S_a_O_2_ and S_v_O_2_ by the following equation:

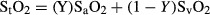
1

where *Y* is the percent contribution of S_a_O_2_ to S_t_O_2_. As the Fick equation requires the arterial and venous blood oxygen difference to be calculated, S_v_O_2_ is required, and can be denoted using S_a_O_2_ and S_t_O_2_:

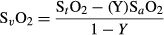
2

Substituting this function into the Fick equation, the following equation can be used to describe CMRO_2_:

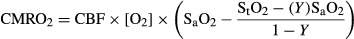
3

The arterial venous function can then be combined into one fraction:

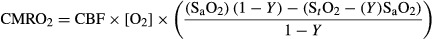
4

This equation can then be simplified as follows, leaving the proportion of S_a_O_2_ contribution to S_t_O_2_ in the denominator only:

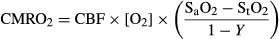
5

Assuming the large vessel hemoglobin concentration and oxygen carrying capacity do not change within the duration of the study, the equation can be simplified by using a ratio to eliminate the proportion of S_a_O_2_ contribution to the NIRS signal. By calculating CMRO_2_ as a ratio, the 1−*Y* denominator would cancel out, and we would get the following equation:

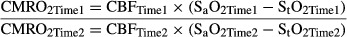
6

Calculating changes in CMRO_2_ as a ratio compared to baseline avoids the problem of the inability to determine the percent contribution of venous and arterial signal to the NIRS S_t_O_2_ signal. We have tested this in equations 3 and 4 using simulated numbers and found that regardless of the proportion of arterial and venous contribution to the NIRS signal, the ratio of the arterial venous oxygen difference calculated using S_t_O_2_ is always be the same.

### Statistics

A doubly repeated measures analysis of covariance (ANCOVA) with orthogonal contrasts was used to evaluate mean differences between the placebo and caffeine interventions across time. This analysis was done to adjust the baseline scores to ensure the groups had similar baseline values. The ANCOVA consisted of mixed linear models which included two fixed within-subject factors that were crossed, intervention (placebo vs. caffeine) and time-of-observation, and the baseline score was used as a covariate. Random intercepts were used to induce the within-subject correlations. Statistical analyses were conducted using SAS version 9.2 (SAS Institute, Cary, NC). All other comparisons were done using a repeated measure ANOVA with a Tukey's test.

## Results

Of the nine subjects recruited, four were unable to complete the experimental protocol. Two subjects could not maintain the appropriate respiratory rate and volume to maintain steady-state hypocapnia, one subject requested to stop the study, and one subject had a poor temporal window for the TCD measurement. The remaining subjects (two females, three males) were included in the analysis.

### Hyperventilation

**Figure 1:**
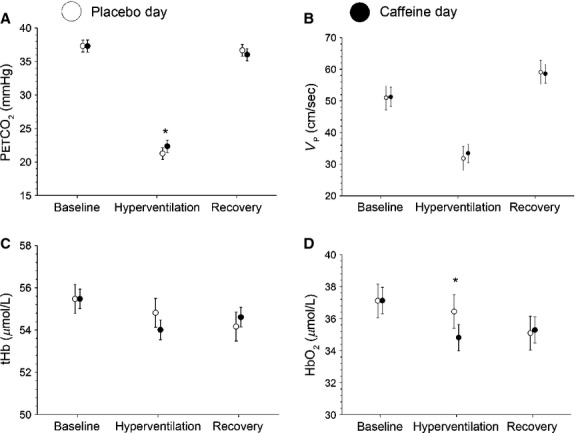
The effect of hypocapnia on different measurement parameters after the applying analysis of covariance (ANCOVA) method to normalize all the data such that the starting baseline is the same between the 2 days. Hyperventilation occurred before the ingestion of caffeine or placebo. Open circles are data from the day caffeine was ingested and closed circles from the day the placebo was ingested. (A) PetCO_2_ declined with controlled hyperventilation. There were significant day to day differences in ΔPetCO_2_ due to hyperventilation. (B) There were significant reductions in transcranial Doppler ultrasound Vp in response to hyperventilation and significant increases during recovery (*P *< 0.01). (C) There was a significant reduction in tHb during hyperventilation (*P *< 0.01). tHb remained significantly lower during recovery. (D) Significant reduction in [HbO_2_] during hyperventilation and recovery (*P *< 0.01). (mean ± 95% CI, *n* = 5). *P < 0.05 versus placebo.

In response to hyperventilation, cerebral S_t_O_2_ and tHb decreased by 2.9 ± 0.6% (*P* < 0.01) (mean ± SEM) and 2.6 ± 0.8% (*P* < 0.01) compared to baseline, respectively. The average decrease in PetCO_2_ in response to hyperventilation was 15.5 ± 1.9 mmHg. Vp was also significantly decreased by 40.0 ± 4.0% compared to baseline (*P* < 0.001). Between the two experimental days, there was a 1.1 mmHg difference in the ΔPetCO_2_ induced by the hyperventilation protocol (Fig.1A), which also resulted in differences in HbO_2_ (Fig.1D), S_t_O_2_, and HHb during the hyperventilation. There were no significant differences in Vp and tHb during hyperventilation between the 2 days (Fig.1B and C). Moreover, there was a 28.6 ± 4.0% reduction in rCMRO_2_ during hyperventilation (*P* < 0.001), which was consistent between 2 days (Fig. 3A).

Compared to baseline, there was an 18.0 ± 3.9% increase in Vp during the recovery period (Fig.1B) despite a similar PetCO_2_ levels (Fig.1A). In contrast, S_t_O_2_ was significantly reduced by 3.1 ± 0.8% (*P* < 0.01) compared to baseline, and was accompanied by a 1.8 ± 0.8% significant decrease in tHb (*P* < 0.05). Similar to Vp, there was a 26.2 ± 4.6% increase in rCMRO_2_ during the recovery phase (Fig. 3A) (*P* < 0.05). This pattern of response was similar between the two experimental days.

### Pharmacological study

Results of the pharmacological study are summarized in Table1. Examining the time course of hemodynamic changes of caffeine, at 60 min after caffeine ingestion, there was a 24.9 ± 3.7% decrease in Vp (*P* < 0.001) in comparison to the precaffeine value. By 60 min, tHb and HbO_2_ decreased by 2.8 ± 1.1% and 3.3 ± 1.3%, respectively, compared to baseline (*P* < 0.01). There were no significant changes in any parameters in the placebo trial (*P* > 0.05).

**Table 1:** Changes in S_t_O_2_, tHb, HbO_2_, and HHb in the pharmacological time course study. Data are presented as mean (SD). These are raw data and have not been normalized by ANCOVA.

Parameters	Placebo			Caffeine		
	Baseline	30 min	60 min	Baseline	30 min	60 min
S_t_O_2_ (%)	65.9 (3)	67.7 (3)	67.9 (2)	63.9 (6)	63.4 (6)	63.4 (5)
tHb (uM)	55.1 (7)	56.3 (7)	56.5 (6)	54.5 (8)	53.5 (8)	53.1 (9)
HbO_2_ (uM)	36.5 (5)	38.2 (6)	38.5 (5)	34.6 (4)	33.6 (4)	33.5 (4)
HHb (uM)	18.7 (2)	18.0 (1)	18.0 (1)	19.9 (5)	19.9 (6)	19.6 (5)
Vp (cm/s)	50.6 (9)	46.4 (6)	45.1 (5)	53.1 (10)	44.8 (13)	43.3 (12)

With respect to precaffeine values, there was a significant decrease in rCMRO_2_ at 30 and 60 min after caffeine ingestion (9.7 ± 3.3% and 16.8 ± 3.6%, respectively, *P* < 0.05). Similar decreases in CMRO_2_ in the placebo group were also observed at 30 and 60 min (11.1 ± 3.2% and 14.1 ± 3.8%, respectively, *P* < 0.05). There was no significant difference in rCMRO_2_ between the caffeine and placebo conditions at any time point (*P* > 0.05) (Fig. 3B).

**Figure 2:**
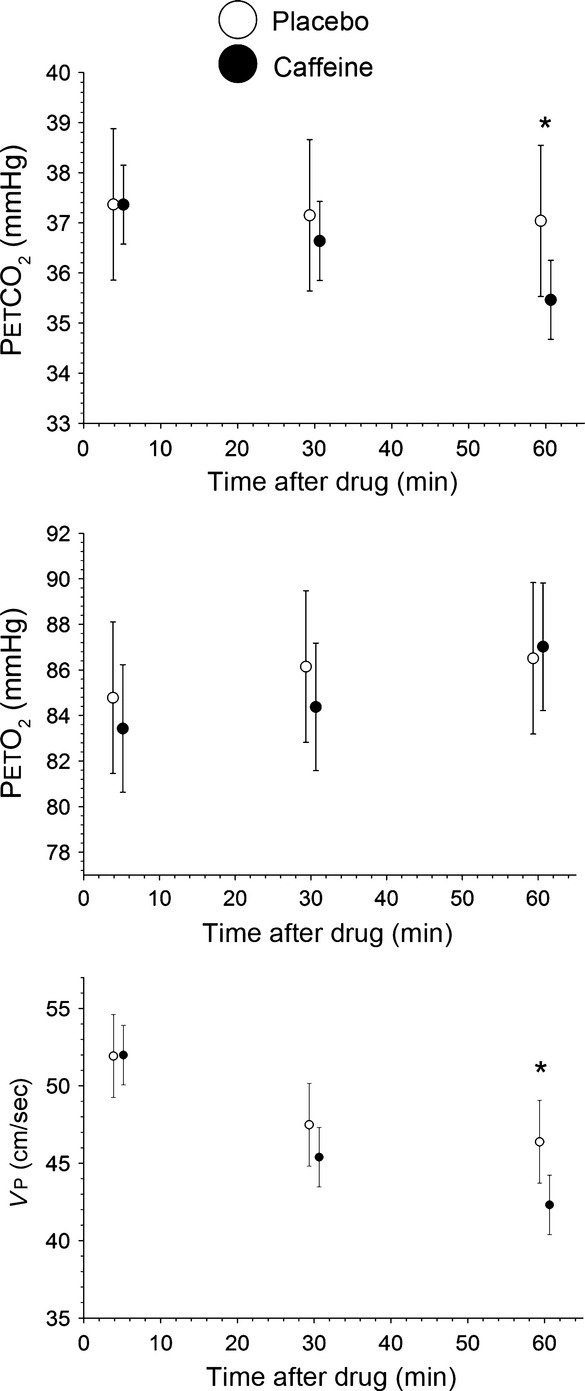
Time course study for the placebo (open circle) and caffeine (solid circle) conditions. The data have been normalized by the ANCOVA method to ensure that the starting values between the caffeine and placebo day are the same. Baseline consisted of 5 min of data. Data from 25 to 35 min make up the 30 min point, and data from 55 to 65 min make up the 60 min point (mean ± 95% CI). *P < 0.05 versus placebo.

As the placebo group showed a trend in the fdNIRS data over the 60 min of the study, we also undertook a doubly repeated analysis of covariance to take into account the effect of the placebo condition. After this analysis, we found significant reductions in S_t_O_2_ (*P* < 0.01), HbO_2_ (*P* < 0.001), and tHb (*P* < 0.001) at 30 min when comparing caffeine to the placebo group (Fig. 4). At 60 min, there were significant differences between caffeine and placebo in PetCO_2_ (*P* < 0.05), Vp (*P* < 0.05), and all NIRS parameters (*P* < 0.05) (Fig.2). In the caffeine condition, relative to placebo, there was a 3.8% decrease in Vp, 4.3% decrease in tHb and 4.0% in S_t_O_2_ decrease at 30 min, and an 8.0% decrease in Vp, 5.5% decrease in tHb and 5.6% decrease in S_t_O_2_ at 60 min.

In order to determine if there is machine-related drift, we measured data from a phantom with known optical properties for 60 min. S_t_O_2_ was 60.7 ± 0.85% at baseline and 60.5 ± 0.72% at 60 min. tHb was 54.98 ± 0.71 μmol/L at baseline, and 56.27 ± 0.56 μmol/L at 60 min.

## Discussion

We used noninvasive multimodal TCD and NIRS imaging to carry out a randomized double-blind, placebo-controlled, cross-over study to investigate the effect of caffeine on cerebral hemodynamics. We used hyperventilation to induce hypocapnia and showed that fdNIRS parameters were sensitive to changes in microvascular hemoglobin concentration. In addition, we showed that 200 mg of caffeine significantly reduces Vp, S_t_O_2_, tHb, and CMRO_2_ compared to placebo.

This cross-over design, coupled with an analysis of covariance statistical modelling yielded a highly efficient analysis of the data. The within-subject intraclass correlation was 0.89 which resulted in a design effect of 0.11. The effective sample size is calculated as the actual sample size divided by the design effect. This implies that a design effect of 0.11 has effectively 9 (1/0.11) times the subject, suggesting that this study has the equivalent power of 90 (45 in each group) subjects randomly assigned to two independent groups.

### The impact of hyperventilation and reduced PetCO_2_

We showed a decline in Vp and S_t_O_2_ in response to hyperventilation-induced hypocapnia, which is in agreement with previous findings (Grote et al. 1981; Clausen et al. 2004; Meng et al. 2012; Alexander et al. 2013). Associated with a decline of MCA Vp, we also observed a significant decline in tHb. The decrease in tHb accompanying the decrease in CBF is in line with previous reports investigating the relationship between large vessel CBF and regional CBV (Leung et al. 2009). PetO_2_ was maintained at a constant level during the hyperventilation paradigm, so hyperventilation-induced physiological changes were independent of PetO_2_.

**Figure 3:**
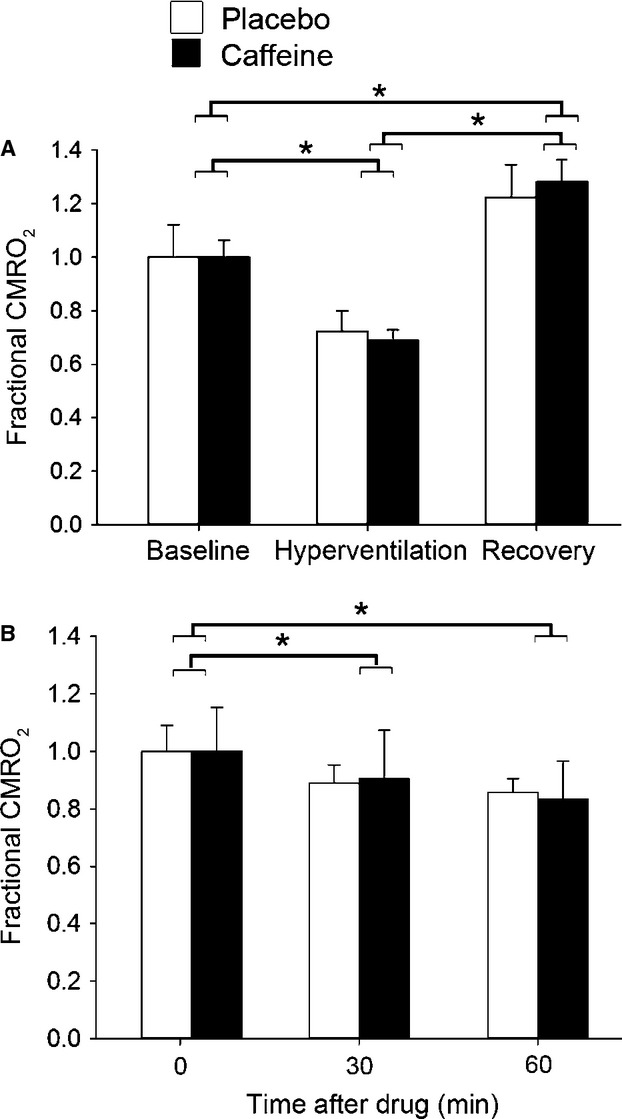
Relative CMRO_2_ values during (A) hypocapnia and (B) time course study. Hyperventilation consisted of 5 min of data for each task (baseline, hyperventilation, recovery). Baseline data from the time course study were the average of 5 min previous to drug ingestion. Data from 25 to 35 min make up the 30 min point, and data from 55 to 65 min make up the 60 min point. These are expressed as the proportion to baseline *P < 0.05 (mean ± SEM)

Changes in MCA blood flow are related to changes in microvessel saturation (Leung et al. 2009), allowing us to combine the TCD measurements with NIRS measurements to estimate CMRO_2_ using the Fick principle. As such, we found that profound hypocapnia caused a 28.6 ± 3.0% decrease in CMRO_2_. This finding is consistent with previous studies reporting hypocapnia may reduce cerebral oxidative metabolism (Grote et al. 1981; Clausen et al. 2004), but contradicts a functional magnetic resonance imaging (fMRI) study suggesting mild hypocapnia does not impact CMRO_2_ (Chen and Pike 2010). Differences in the magnitude of decrease in PetCO_2_ with the hyperventilation may explain this discrepancy. (Chen and Pike 2010) used hyperventilation to induce a ∼5 mmHg decrease in PetCO_2_, while we used hyperventilation and dynamic end-tidal forcing function to maintain a ∼15.5 mmHg decrease in PetCO_2_. Consequently, in the current study, it is likely that the ∼40% decrease in CBF during profound hypocapnia cannot be fully compensated for by the increase in oxygen extraction, causing a net decrease in CMRO_2_. Interestingly, during the recovery period after hyperventilation, we found CMRO_2_ as well as Vp were elevated compared to baseline levels. Moreover, the magnitude of CMRO_2_ decrease during hyperventilation was similar to the magnitude of CMRO_2_ increase during recovery (28.6% vs. 26.2%). This would suggest there is a compensatory effect for the reduced oxygen metabolism during the hyperventilation phase. This response pattern to hyperventilation for Vp and CMRO_2_ was consistent between the two experimental days (Figs.1, 3A).

### The impact of caffeine on cerebral hemodynamics

**Figure 4:**
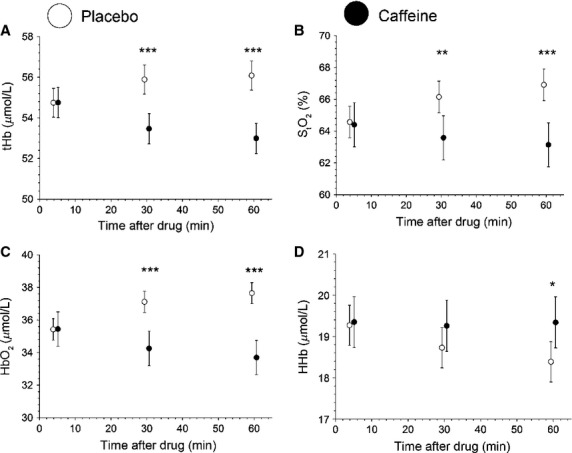
Time course data of NIRS parameters after ANCOVA normalization. Solid circle denotes caffeine and open circle denotes placebo. Baseline consisted of 5 min of data. Data from 25 to 35 min make up the 30 min point, and data from 55 to 65 min make up the 60 min point. Significance was determined between placebo and caffeine trial. *P < 0.05 versus placebo; **P < 0.01 versus placebo; ***P < 0.001 versus placebo.

There was a 24.9 ± 3.7% reduction in Vp as well as a 2.8 ± 1.1% reduction tHb in the caffeine condition compared to precaffeine values. The drop in Vp is in agreement with previous reports using both TCD (MCA) and magnetic resonance imaging (MRI) arterial spin labeling (motor cortex) (Lunt et al. 2004; Griffeth et al. 2013), showing a 22% and 26.9% decrease after 200 mg of caffeine ingestion, respectively. The drop in Vp with caffeine is in agreement with the decline in tHb that we (Fig.4) and others (Higashi et al. 2004) have observed.

In time course studies where the subject is quiet for an hour or more, there may be changes in ventilation and, thus changing arterial blood gases. If changes in PetCO_2_ occur, this would impact Vp as arterial PCO_2_ (PaCO_2_) is a major modulator of CBF. PetCO_2_ decreased by 1.5 mmHg in the caffeine group, which would typically result in a 4–5% decrease in Vp (Ide et al. 2003). We observed a 24.9% decrease in Vp. Therefore, although caffeine may have caused changes in ventilation which could contribute to the observed decline in Vp, caffeine had a much larger impact in decreasing blood flow.

Both caffeine and placebo ingestion resulted in a reduction in CMRO_2_ at 30 and 60 min (Fig.3B). The lack of significant difference in CMRO_2_ between caffeine and placebo indicates that caffeine does not change CMRO_2_. If neurovascular coupling is constant, then CMRO_2_, perfusion and oxygen extraction should be linked such that if CMRO_2_ decreases, there should be a decrease in CBF and an increase in OEF (Kida et al. 2007). This would result in a decrease in HbO_2_. We observed a decrease in Vp and a decrease in tHb—consistent with reduced overall blood flow as well as local microvascular flow. We also observed a decrease in HbO_2_ and S_t_O_2_ on the caffeine day with respect to placebo. These changes are all consistent with reduced CMRO_2_. Thus, either we did not have sufficient power to detect a decline in CMRO_2_ that exists, or there is a change in neurovascular coupling with caffeine.

Existing literature regarding caffeine-induced changes in rCMRO_2_ is inconclusive. Perthen et al. (2008) did not observe any change in rCMRO_2_ 60 min after ingestion of 200 mg of caffeine, while more recently the same group reported a 22% increase in rCMRO_2_ after 60 min of 200 mg of caffeine ingestion (Griffeth et al. 2013). However, the same paper reported a concurrent 27% decrease in CBF. We estimate that, with this reduced CBF, the OEF would need to increase by 67% for such an increase in rCMRO_2_. As this is unlikely to occur, it raises doubts about their observed CMRO_2_ changes.

Interestingly, we noticed a drift in the NIRS and TCD parameters, which resulted in a significant decline in CMRO_2_ over the period of an hour. It is possible that there are confounding effects that cause drift in the NIRS data which are not related to brain physiology. For instance, the probe contact may change. We have data where subjects are alert, and there is <1% change in saturation. In order to rule out machine drift, we undertook a 1 h study on a calibration phantom. This showed no appreciable drift, indicating that the changes are not due to machine-related errors. TCD measurements of MCA blood flow in previous studies have also shown consistency during measurements of up to 1 h, where subjects were alert (Vantanajal et al. 2007). It has been shown that measurements of CMRO_2_ several hours apart yield consistent values (Bartlett et al. 1988). We showed that that NIRS and TCD parameters will drift if subjects sit quietly for an hour in a dimly lit room with no external stimuli. This trend in the placebo group emphasizes the use of a placebo in long experiments. The use of a placebo will reduce the impact of any instrumental drift. Had we not included a placebo group, we would have underestimated the impact of caffeine on brain hemodynamics.

### Limitations

There are limitations associated with the use of the NIRS technique in our study. A significant portion of the NIRS signal is passing through the scalp, skull and cerebrospinal fluid (Gagnon et al. 2011), which will contribute to the NIRS values. Since caffeine decreases blood flow to skeletal muscle (Salahdeen and Alada 2009), it is possible that it will also cause a decline in blood flow within the skull and scalp, thus contaminating the NIRS signal coming from the brain. However, because the NIRS probe is tightly pressed against the forehead, there should be very little blood in the skin and scalp, which will lessen their impact. In addition, there is little reason to expect any changes in blood flow of the skin while being quietly seated. Lastly, to account for the physiological effects of being quietly seated, we incorporated a placebo control into our experiment, which allows us to identify the sole effect of caffeine.

Besides contamination of the NIRS signal, the calculated CMRO_2_ will also have inaccuracies. TCD measures MCA blood flow velocity, while NIRS measures microvascular oxygenation. Although there is a good relationship between large vessel blood flow and microvessel data (Leung et al. 2009), changes in large vessel CBF will not completely reflect changes in small vessel CBF. The discrepancy between these two areas of the cerebral vasculature will affect CMRO_2_ calculation. To reduce the impact of this discrepancy, we calculated CMRO_2_ as a fraction of the baseline, and not absolute CMRO_2_. Calculating CMRO_2_ in this manner reduces the impact of other variables, such as the difference between large and microvessel hematocrit, that may affect CMRO_2_.

Several studies have suggested that TCD may underestimate the CBF changes under certain conditions due to changes in MCA diameter (Wilson et al. 2011; Willie et al. 2012). To solve this problem, the TCD system we used continuously assesses, and outputs, the total power of the received Doppler signal, which has been shown to be proportional to the cross-sectional area of the insonated artery (Poulin and Robbins 1996; Hatab et al. 1997). Within the hyperventilation component of the study, examination of the TCD power signal revealed no significant difference between baseline, hyperventilation and recovery. Furthermore, there was no significant difference in the TCD power signal between the different time points in the pharmacological time course study. This suggests the changes observed in peak blood velocity (i.e., Vp) within the current study are reflective of changes in CBF.

Hypocapnia will cause changes in blood CO_2_ and subsequently pH, thereby affecting the oxygen dissociation curve and oxygen diffusion. In addition, under hypocapnia, the venous blood oxygenation is no longer in the same function of O_2_ partial pressure as normal ventilation condition, which may impact NIRS oxygenation measurements. Fortunately, the effect of pH and blood CO_2_ on the oxygen dissociation curve should be accounted for by the NIRS measurement itself, which measures hemoglobin saturation. Hypocapnia would be expected to impact the oxyhemoglobin dissociation curve of the total vasculature and so have the same relative impact on the microvascular and venous blood.

## Conclusion

In conclusion, we demonstrated that a combined NIRS and TCD approach is sensitive at detecting physiologically and pharmacologically induced hemodynamic changes and so could be useful at individualizing drug responses. We showed that profound hypocapnia significantly reduced brain oxygenation and CMRO_2_. We conducted a double-blinded, placebo-controlled, cross-over experiment to study caffeine's hemodynamic effects, demonstrating that caffeine reduces CBF through the MCA, as well as S_t_O_2_ and CBV within the frontal lobe. By combining the fdNIRS and the TCD data, we also showed that caffeine may not significantly alter CMRO_2_ compared to placebo. Our placebo data show that 60 min of inactivity will impact brain hemodynamics, suggesting that the use of a placebo is important in pharmacological studies that involve a long duration of data collection. This multimodal imaging technique could prove to be very useful in studying the hemodynamic and metabolic changes related to the effects of pharmacological substances.
